# On Optimal Backward Perturbation Analysis for the Linear System with Skew Circulant Coefficient Matrix

**DOI:** 10.1155/2013/707381

**Published:** 2013-11-28

**Authors:** Juan Li, Zhaolin Jiang, Nuo Shen, Jianwei Zhou

**Affiliations:** ^1^Department of Mathematics, Linyi University, Linyi, Shandong 276000, China; ^2^Department of Mathematics, Shandong Normal University, Ji'nan, Shandong 250014, China

## Abstract

We first give the style spectral decomposition of a special skew circulant matrix *C* and then get the style decomposition of arbitrary skew circulant matrix by making use of the Kronecker products between the elements of first row in skew circulant and the special skew circulant *C*. Besides that, we obtain the singular value of skew circulant matrix as well. Finally, we deal with the optimal backward perturbation analysis for the linear system with skew circulant coefficient matrix on the base of its style spectral decomposition.

## 1. Introduction

 A skew circulant matrix with the first row (*a*_1_, *a*_2_,…, *a*_*n*_) is a square matrix of the form

(1) $$\begin{array}{c} {\left(\begin{array}{ccccc} a_{1} & a_{2} & \cdots & a_{n-1} & a_{n}\\ -a_{n} & a_{1} & a_{2} & \cdots & a_{n-1}\\ \vdots & \ddots & \ddots & \ddots & \vdots \\ -a_{3} & \cdots & -a_{n} & a_{1} & a_{2}\\ -a_{2} & -a_{3} & \cdots & -a_{n} & a_{1} \end{array}\right)}_{n\times n}, \end{array}$$

denoted by SCirc  (*a*_1_, *a*_2_,…, *a*_*n*_).

Skew circulant matrices have important applications in various disciplines including image processing, signal processing, solving Toeplitz matrix problems, and preconditioner. The skew circulant matrices are considered as preconditioners for linear-multistep-formulae (LMF-) based ordinary differential equations (ODEs) codes; Hermitian and skew-Hermitian Toeplitz systems are considered in [1–4]. Lyness and Sörevik [5] employed a skew circulant matrix to construct *s*-dimensional lattice rules. Spectral decompositions of skew circulant and skew left circulant matrices are discussed in [6]. Akhondi and Toutounian [7] presented a new iteration method for the numerical solution of Hermitian positive definite Toeplitz systems of linear equations. Narasimha [8] believed that the linear convolution required in block filtering can be decomposed into a sum of skew-circulant convolutions and such convolutions can be realized efficiently with half-length complex transforms when the signals are real. Liu and Vaidyanathan [9] presented a new family of normal form state-space structures, the method used allows people to synthesize in normal form, most IIR transfer functions, and the state transition matrices involved are either circulant or skew circulant matrices. Vaidyanathan and Pal [10] examined a case where two arrays are generated by matrices that are adjugates of each other; in this case, it is possible to obtain a dense rectangular tiling of the 2*D* frequency plane from a pair of coarse 2*D* DFT filter banks; the special case where the adjugate pairs are generated by skew circulant matrices has some advantages, which are examined in detail. An additional convolution-multiplication property for the skew-circulant convolution operation *y* = *hⓈx* = *H*_*s*_ *x*, where *H*_*s*_ is a skew-circulant matrix; besides, skew-circulant convolution is the underlying form of convolution in half of the 40 cases of symmetric convolution, and the convolution is an extension of a result Vernet's [11], Foltz and Welsh provided the convolution performed between *h* and *x* is skew-circulant rather than circulant in [12].

Liu and Guo [13] gave the optimal backward perturbation analysis for a linear system with block circulant coefficient matrix. The optimal backward perturbation bound for underdetermined systems is studied by J.-G. Sun and Z. Sun in [14]. Some new theorems generalizing a result of Oettli and Prager are applied to the a posteriori analysis of the compatibility of a computed solution to the uncertain data of a linear system by Rigal and Gaches in [15].

In this paper, we first give the style spectral decomposition of a special skew circulant matrix *C* and then get the style spectral decomposition of arbitrary skew circulant matrix by making use of Kronecker products between the elements of first row in skew circulant and the special skew circuant *C*. Besides that, we obtain the singular value of skew circulant matrix as well. Finally, we deal with the optimal backward perturbation analysis for the linear system with skew circulant coefficient matrix on the base of its style spectral decomposition. 

## 2. The Style Spectral Decomposition of Skew Circulant Matrix

### 2.1. Style Spectral Decomposition of a Special Skew Circulant Matrix

 Let

(2) $$\begin{array}{c} C=\left(\begin{array}{ccccc} 0 & 1 & 0 & \cdots & 0\\ 0 & 0 & 1 & \cdots & 0\\ \vdots & \ddots & \ddots & \ddots & \vdots \\ 0 & \cdots & 0 & 0 & 1\\ -1 & 0 & \cdots & 0 & 0 \end{array}\right). \end{array}$$

Some properties of this matrix are given in the following theorem.

**Lemma 1**

(1) The eigenvalues of matrix *C* are

(3) $$\begin{array}{c} \lambda_{j}=e^{i((2j-1)/n)\pi },\;j=1,2,\ldots ,n. \end{array}$$

(2) If *n* is even, the matrix *C* has no real eigenvalue and

(4) $$\begin{array}{c} \lambda_{j}={\bar{\lambda }}_{n+1-j},\;j=1,2,\ldots ,\frac{n}{2}. \end{array}$$

The basis of the associated two-dimensional invariant subspace can be taken as

(5) $$\begin{array}{c} x_{j}^{\left(1\right)}=\left(\begin{array}{c} 1\\ \cos\theta_{j}\\ \vdots \\ \cos\left(n-1\right)\theta_{j} \end{array}\right),\\ x_{j}^{\left(2\right)}=\left(\begin{array}{c} 0\\ \sin\theta_{j}\\ \vdots \\ \sin\left(n-1\right)\theta_{j} \end{array}\right),\\ \theta_{j}=\frac{2j-1}{n}\pi ,\;j=1,2,\ldots ,\frac{n}{2}. \end{array}$$

(3) If *n* is odd, the matrix *C* has only one real eigenvalue *λ*_(*n*+1)/2_ = −1, and the associated eigenvector is

(6) $$\begin{array}{c} x_{(n+1)/2}=\left(\begin{array}{c} 1\\ -1\\ 1\\ -1\\ \vdots \\ 1 \end{array}\right),\\ \lambda_{j}={\bar{\lambda }}_{n+1-j},\;j=1,2,\ldots ,\frac{n-1}{2}. \end{array}$$

The basis of the associated two-dimensional invariant subspace can be taken as

(7) $$\begin{array}{c} x_{j}^{\left(1\right)}=\left(\begin{array}{c} 1\\ \cos\theta_{j}\\ \vdots \\ \cos\left(n-1\right)\theta_{j} \end{array}\right),\\ x_{j}^{\left(2\right)}=\left(\begin{array}{c} 0\\ \sin\theta_{j}\\ \vdots \\ \sin\left(n-1\right)\theta_{j} \end{array}\right),\\ \theta_{j}=\frac{2j-1}{n}\pi ,\;j=1,2,\ldots ,\frac{n-1}{2}. \end{array}$$

Specially, if *n* is even, then *x*_*j*_^(1)^ and *x*_*j*_^(2)^ span the two-dimensional invariant subspace associated with *λ*_*j*_ and $\bar{\lambda_{j}}$.

**Lemma 2**

(1)  *x*_*j*_^(1)^ and *x*_*j*_^(2)^ are orthogonal.

(2)  *x*_*j*_^(*l*)^ and *x*_*k*_^(*s*)^ are orthogonal (*j* ≠ *k*) (*l*, *s* = 1,2).

(3) Also ${||x_{(n+1)/2}||}_{2}=\sqrt{n}$, ${||x_{j}^{\left(1\right)}||}_{2}={||x_{j}^{\left(2\right)}||}_{2}=\sqrt{n/2}$,

(8) $$\begin{array}{c} {||\left(\begin{array}{c} 1\\ \lambda_{j}\\ \vdots \\ \lambda_{j}^{n-1} \end{array}\right)||}_{2}=\sqrt{n}. \end{array}$$

Let *Q* = [*q*_1_, *q*_2_,…, *q*_*n*_], where

(9) $$\begin{array}{c} q_{2j-1}=\sqrt{\frac{2}{n}}x_{j}^{\left(1\right)},\;q_{2j}=\sqrt{\frac{2}{n}}x_{j}^{\left(2\right)}\;\left(j=1,2,\ldots ,\left[\frac{n}{2}\right]\right),\\ q_{n}=\sqrt{\frac{1}{n}}\left(\begin{array}{c} 1\\ -1\\ 1\\ -1\\ \vdots \\ 1 \end{array}\right)\;\left(n\;\;\text{is}\;\;\text{odd}\right). \end{array}$$

Then *Q* is an orthogonal matrix, and if *n* is even,

(10) $$\begin{array}{c} C=Q\left(\begin{array}{cccc} C_{1} & & & \\ & C_{2} & & \\ & & \ddots & \\ & & & C_{n/2} \end{array}\right)Q^{T}, \end{array}$$

where $C_{j}=\left(\begin{array}{cc} \cos\theta_{j} & \sin\theta_{j}\\ -\sin\theta_{j} & \cos\theta_{j} \end{array}\right)\;\;(j=1,2,\ldots ,n/2)$.

 When *n* is odd,

(11) $$\begin{array}{c} C=Q\left(\begin{array}{cccc} C_{1} & & & \\ & \ddots & & \\ & & C_{(n-1)/2} & \\ & & & -1 \end{array}\right)Q^{T}, \end{array}$$

where $C_{j}=\left(\begin{array}{cc} \cos\theta_{j} & \sin\theta_{j}\\ -\sin\theta_{j} & \cos\theta_{j} \end{array}\right)\;(j=1,2,\ldots ,(n-1)/2)$.

 In fact, (10) and (11) are the style spectral decomposition of the matrix *C*. 

### 2.2. The Style Spectral Decomposition of the Skew Circulant Matrix

We have

(12) A=(a1a2⋯an−1an−ana1a2⋯an−1⋮⋱⋱⋱⋮−a3⋯−ana1a2−a2−a3⋯−ana1)=a1·C0+a2·C1+⋯+an·Cn−1=a1·(QC00QT)+⋯+an·(QC0n−1QT)=Q(a1·C00)QT+⋯+Q(an·C0n−1)QT

(13) $$\begin{array}{c} =Q\left(\sum_{k=1}^{n}a_{k}\cdot C_{0}^{k-1}\right)Q^{T}, \end{array}$$

where

(14) $$\begin{array}{c} C_{0}=\left(\begin{array}{cccc} C_{1} & & & \\ & C_{2} & & \\ & & \ddots & \\ & & & C_{n/2} \end{array}\right) \end{array}$$

(*n* is even, the same case as (10)),

(15) $$\begin{array}{c} C_{0}=\left(\begin{array}{cccc} C_{1} & & & \\ & \ddots & & \\ & & C_{(n-1)/2} & \\ & & & -1 \end{array}\right) \end{array}$$

(*n* is odd, the same case as (11)).

Noticing that *Q* is an orthogonal matrix, hence (13) is the style spectral decomposition of the matrix *A*.

The following are the computation formulae of the factors in (13):

(16) $$\begin{array}{c} C_{j}^{k}=\left(\begin{array}{cc} \cos k\theta_{j} & \sin k\theta_{j}\\ -\sin k\theta_{j} & \cos k\theta_{j} \end{array}\right). \end{array}$$

Hence, when *n* is even,

(17) $$\begin{array}{c} \sum_{k=1}^{n}a_{k}\cdot C_{0}^{k-1}=\left(\begin{array}{cccc} {\tilde{A}}_{1} & & & \\ & {\tilde{A}}_{2} & & \\ & & \ddots & \\ & & & {\tilde{A}}_{n/2} \end{array}\right), \end{array}$$

where, for arbitrary *j* = 1,2,…, *n*/2,

(18) $$\begin{array}{c} {\tilde{A}}_{j}=\left(\begin{array}{cc} \sum_{k=1}^{n}a_{k}\cos k\theta_{j} & \sum_{k=1}^{n}a_{k}\sin k\theta_{j}\\ -\sum_{k=1}^{n}a_{k}\sin k\theta_{j} & \sum_{k=1}^{n}a_{k}\cos k\theta_{j} \end{array}\right). \end{array}$$

When *n* is odd,

(19) $$\begin{array}{c} \sum_{k=1}^{n}a_{k}\cdot C_{0}^{k-1}=\left(\begin{array}{cccc} {\tilde{A}}_{1} & & & \\ & \ddots & & \\ & & {\tilde{A}}_{(n-1)/2} & \\ & & & A_{*} \end{array}\right), \end{array}$$

where ${\tilde{A}}_{j}$ is defined by (18), and

(20) $$\begin{array}{c} A_{*}={\left(-1\right)}^{k}\sum_{k=1}^{n}a_{k}. \end{array}$$

Hence the style spectral decomposition of the matrix *A* is

(21) $$\begin{array}{c} A=Q\left(\begin{array}{cccc} \sum_{k=1}^{n}a_{k}C_{1}^{k-1} & & & \\ & \sum_{k=1}^{n}a_{k}C_{2}^{k-1} & & \\ & \ddots & & \\ & & \ddots & \end{array}\right)Q^{T}. \end{array}$$

## 3. The Structured Perturbation Analysis

 In this section we give the structured perturbation analysis for linear systems with skew circulant coefficient matrix.

### 3.1. Condition Number and Relative Error of Linear Skew Circulant Equation System

Consider the following:

(22) $$\begin{array}{c} Ax=b, \end{array}$$

where *A* is defined in (2).

From (13), we know that the style spectral decomposition of the matrix *A* is

(23) $$\begin{array}{c} A=Q\left(\begin{array}{ccc} A_{11} & & \\ & \ddots & \\ & & A_{tt} \end{array}\right)Q^{T}. \end{array}$$

When *n* is even and *t* = *n*/2,

(24) $$\begin{array}{c} A_{jj}=\sum_{k=1}^{n}a_{k}\left(\begin{array}{cc} \cos\left(k-1\right)\theta_{j} & \sin\left(k-1\right)\theta_{j}\\ -\sin\left(k-1\right)\theta_{j} & \cos\left(k-1\right)\theta_{j} \end{array}\right),\\ \theta_{j}=\frac{2j-1}{n}\pi ,\;j=1,2,\ldots ,t. \end{array}$$

When *n* is odd and *t* = (*n* − 1)/2 + 1, *A*_*jj*_ is defined in (24) (*j* = 1,2,…, (*n* − 1)/2) and

(25) $$\begin{array}{c} A_{tt}={\left(-1\right)}^{k}\sum_{k=1}^{n}a_{k}. \end{array}$$

**Lemma 3**

*A* is an invertible matrix if and only if *f*(*ω*_*j*_) ≠ 0 (*j* = 1,2,…, *n*), where

(26) $$\begin{array}{c} f\left(\omega_{j}\right)=\sum_{k=1}^{n}a_{k}\omega_{j}^{k-1},\;\omega_{j}=e^{i((2j-1)/n)\pi },\;\;j=1,2,\ldots ,n. \end{array}$$

Let

(27) $$\begin{array}{c} \sigma_{j}=\left|f\left(\omega_{j}\right)\right|,\;j=1,2,\ldots ,n,\\ 𝒦=\frac{\max\left\{\sigma_{j}\right\}}{\min\left\{\sigma_{j}\right\}}. \end{array}$$

**Remark 4**

The singular values of matrix *A* are *σ*_1_, *σ*_2_,…, *σ*_*n*_.

The proof of Lemma 3 and Remark 4 is given in the following:

(28) $$\begin{array}{c} A=\sum_{k=1}^{n}a_{k}C^{k-1}. \end{array}$$

Consequently, the spectral decomposition of the matrix *A* (by using the complex style spectral decomposition of $C=Q_{0}\left(\begin{array}{ccc} \omega_{1} & & \\ & \ddots & \\ & & \omega_{n} \end{array}\right)Q_{0}^{*}$) is

(39) $$\begin{array}{c} A=Q_{0}\left(\begin{array}{cccc} f\left(\omega_{1}\right) & & & \\ & f\left(\omega_{2}\right) & & \\ & & \ddots & \\ & & & f\left(\omega_{n}\right) \end{array}\right)Q_{0}^{*}, \end{array}$$

where *Q*_0_ is a unitary matrix.

Let Δ*A*, Δ*b* be the perturbation of the coefficient matrix *A* and vector *b*, respectively, where

(30) $$\begin{array}{c} \Delta A=\left(\begin{array}{ccccc} \delta a_{1} & \delta a_{2} & \cdots & \delta a_{n-1} & \delta a_{n}\\ -\delta a_{n} & \delta a_{1} & \delta a_{2} & \cdots & \delta a_{n-1}\\ \vdots & \ddots & \ddots & \ddots & \vdots \\ -\delta a_{3} & \cdots & -\delta a_{n} & \delta a_{1} & \delta a_{2}\\ -\delta a_{2} & -\delta a_{3} & \cdots & -\delta a_{n} & \delta a_{1} \end{array}\right). \end{array}$$

Let

(31) $$\begin{array}{c} \hat{A}=A+\Delta A,\;\;\hat{b}=b+\delta b,\\ \hat{f}\left(\omega_{j}\right)=\sum_{k=1}^{n}\left(a_{k}+\delta a_{k}\right)\omega_{j}^{k-1}. \end{array}$$

If

(32) $$\begin{array}{c} \sum_{k=1}^{n}\left|\delta a_{k}\right|<\underset{1\leq j\leq n}{\min}\left\{\sigma_{j}\right\}, \end{array}$$

then

(33) |f^(ωj)|≥|∑k=1nakωjk−1|−∑k=1n|δak||ωj|k−1≥min⁡1≤j≤n{σj}−∑k=1n|δak|>0.

Hence $\hat{A}$ is an invertible matrix. Let

(34) $$\begin{array}{c} \sigma_{\min}=\underset{1\leq j\leq n}{\min}\left\{\sigma_{j}\right\},\;\;\Delta =\sum_{k=1}^{n}\left|\delta a_{k}\right|. \end{array}$$

By *Ax* = *b* and $\hat{A}\hat{x}=\hat{b}$, we get

(35) x^−x=A^−1b^−A−1b=A^−1(b+δb)−A−1b=A^−1δb+(A^−1−A−1)b=A^−1δb+(A^−1−A−1)Ax=A^−1δb+A^−1(A−A^)x,||x^−x||2≤||A^−1||2||δb||2+||A^−1||2||A^−A||2||x||2≤||δb||2σmin⁡−Δ+||A^−A||2||x||2σmin⁡−Δ,||x^−x||2||x||2≤||δb||2(σmin⁡−Δ)||x||2+||A^−A||2σmin⁡−Δ≤||A||2σmin⁡−Δ(||δb||2||b||2+||A^−A||2||A||2),

where

(36) $$\begin{array}{c} {||A||}_{2}=\underset{1\leq j\leq n}{\max}\left\{\sigma_{j}\right\}. \end{array}$$

Notice that $\hat{A}-A=\Delta A$ is a skew circulant matrix, and ${||A-\hat{A}||}_{2}=|-1|{||\hat{A}-A||}_{2}={||\hat{A}-A||}_{2}$. So we get

(37) ||A^−A||2=max⁡1≤j≤n|∑k=1nδakωjk−1|≤∑k=1n|δak||ωj|k−1=∑k=1n|δak|=Δ.

Hence we have the following theorem.

**Theorem 5**

Let *A*, $\hat{A}$, *δb*, Δ, and *σ*_min⁡_ be defined as above. If Δ < *σ*_min⁡_, then

(38) $$\begin{array}{c} \frac{{||\hat{x}-x||}_{2}}{{||x||}_{2}}\leq \frac{\sigma_{\max}}{\sigma_{\min}-\Delta }\left(\frac{{||\delta b||}_{2}}{{||b||}_{2}}+\frac{\Delta }{\sigma_{\max}}\right), \end{array}$$

where

(39) $$\begin{array}{c} \sigma_{\max}={||A||}_{2}. \end{array}$$

**Remark 6**

From (38) and (39), the condition number of the skew circulant system can be defined as *𝒦* = max⁡{*σ*_*j*_}/min⁡{*σ*_*j*_}. It is easily computed, as well as the bound of perturbation (38).

### 3.2. Optimal Backward Perturbation Bound of the Linear Skew Circulant Equation System

 Let $\hat{x}$ be an approximate solution to *Ax* = *b* and let

(40) $$\begin{array}{c} \Omega \equiv \left\{\left(\Delta A,\Delta b\right)\;|\;\left(A+\Delta A\right)\hat{x}=b+\Delta b\right\},\\ \eta \left(\hat{x}\right)\equiv \underset{\left(\Delta A,\Delta b\right)\in \Omega }{\inf}||\left[\Delta A,\Delta b\right]||,\\ \left(A+\Delta A\right)\hat{x}=b+\Delta b \end{array}$$

which is equivalent to

(41) $$\begin{array}{c} \left(\Delta A,\Delta b\right)\left(\begin{array}{c} \hat{x}\\ -1 \end{array}\right)=b-A\hat{x}. \end{array}$$

Due to [15], we have

(42) $$\begin{array}{c} \eta \left(\hat{x}\right)=\frac{{||b-A\hat{x}||}_{2}}{\sqrt{1+{||\hat{x}||}_{2}^{2}}}\left(||\cdot ||\;\;\text{being}\;\;\text{unitary}\;\;\text{invariant}\;\;\mathrm{norm}\right). \end{array}$$

If the recycling property of *A* is not utilized in the algorithm in forming $\hat{x}$, then $\eta (\hat{x})$ can be used to estimate the backward stability for this algorithm.

Let $\hat{x}$ be an approximate solution to *Ax* = *b*, where *A* is defined in (2):

(43) Ω≡{(ΔA,Δb) ∣ (A+ΔA)x^=b+Δb, ΔA is a skew circulant matrix}η(x^)≡inf⁡(ΔA,Δb)∈Ω{||(ΔA,Δb)||F}.

Then *Ω* ≠ *ϕ* (such that Δ*A* = 0 is a skew circulant matrix, and $\Delta b=A\hat{x}-b$) and

(44) $$\begin{array}{c} \eta^{2}\left(\hat{x}\right)=\underset{\left(\Delta A,\Delta b\right)\in \Omega }{\inf}\left\{{||\Delta A||}_{F}^{2}+{||\Delta A\hat{x}+A\hat{x}-b||}_{F}^{2}\right\}. \end{array}$$

Since

(45) $$\begin{array}{c} \Delta A=\sum_{k=1}^{n}\delta a_{k}C^{k-1}, \end{array}$$

so

(46) $$\begin{array}{c} {||\Delta A||}_{F}^{2}=n\sum_{k=1}^{n}{\left(\delta a_{k}\right)}^{2}. \end{array}$$

Besides that, we can get

(47) ||ΔAx^+Ax^−b||F2 =||QC∗QTx^+Ax^−b||F2 =||(∑k=1nδakC1k−1x1(0)⋮∑k=1nδakCtk−1xt(0))−r0||F2 =||G0(δa1⋮δan)−r0||F2,

where

(48) $$\begin{array}{c} C_{*}=\left(\begin{array}{ccc} \sum_{k=1}^{n}\delta a_{k}C_{1}^{k-1} & & \\ & \ddots & \\ & & \sum_{k=1}^{n}\delta a_{k}C_{t}^{k-1} \end{array}\right),\\ r_{0}=Q^{T}\left(b-A\hat{x}\right),\;\;Q^{T}\hat{x}=\left(\begin{array}{c} x_{1}^{\left(0\right)}\\ \vdots \\ x_{t}^{\left(0\right)} \end{array}\right),\\ G_{0}=\left(\begin{array}{ccc} C_{1}^{0}x_{1}^{\left(0\right)} & \cdots & C_{1}^{n-1}x_{1}^{\left(0\right)}\\ \vdots & \ddots & \vdots \\ C_{t}^{0}x_{t}^{\left(0\right)} & \cdots & C_{t}^{n-1}x_{t}^{\left(0\right)} \end{array}\right). \end{array}$$

Let

(49) $$\begin{array}{c} f\left(\delta a_{1},\ldots ,\delta a_{n}\right)=n\sum_{k=1}^{n}{\left(\delta a_{k}\right)}^{2}+{||G_{0}\left(\begin{array}{c} \delta a_{1}\\ \vdots \\ \delta a_{n} \end{array}\right)-r_{0}||}_{F}^{2}; \end{array}$$

then

(50) $$\begin{array}{c} \frac{\partial f}{\partial \delta a_{k}}=0 \end{array}$$

which is equivalent to

(51) $$\begin{array}{c} \left(2nI_{n}+2G_{0}^{T}G_{0}\right)\left(\begin{array}{c} \delta a_{1}\\ \vdots \\ \delta a_{n} \end{array}\right)-2G_{0}^{T}r_{0}=0.\\ \frac{\partial^{2}f}{\partial {\left(\delta a_{k}\right)}^{2}}=2nI_{n}+2G_{0}^{T}G_{0}>0, \end{array}$$

Hence the *f* is a convex function about (*δa*_1_,…, *δa*_*n*_), and the point of minimal value is

(52) $$\begin{array}{c} \left(\begin{array}{c} \delta a_{1}\\ \vdots \\ \delta a_{n} \end{array}\right)={\left(nI_{n}+G_{0}^{T}G_{0}\right)}^{-1}G_{0}^{T}r_{0}. \end{array}$$

Substituting it back into (49), we can get the following.

**Theorem 7**

One has

(53) η(x^)2=nr0TG0(nIn+G0TG0)−2G0Tr0 +||[G0(nIn+G0TG0)−1G0T−In]r0||F2.

Let *G*_0_ = *U*ΣV* be the singular value decomposition of *G*_0_, where *U* and *V* are unitary (in fact, *U* and *V* can be real orthogonal), Σ = diag⁡(*σ*_1_′,…, *σ*_*n*_′), and *σ*_*j*_′ ≥ 0  (*j* = 1,2,…, *n*). Hence we have

(54) η(x^)2=nr0TUΣVT(nIn+Σ2)−2VΣUTr0+||[UΣVT(nIn+Σ2)−1VΣUT−In]r0||F2=nr1TΣ(nIn+Σ2)−2Σr1+||[Σ(nIn+Σ2)−1Σ−In]r0||F2=nr1TΣ(nIn+Σ2)−2Σr1+||[Σ(nIn+Σ2)−1Σ−In]r1||F2=nr1TΣ(nIn+Σ2)−2Σr1+n2r1T(nIn+Σ2)−2r1=r1T(d1⋱dn)r1,

where *r*_1_ = *U* ^*T*^ *r*_0_, and *d*_*j*_ = (*nσ*_*j*_^′2^ + *n* ^2^)/(*n* + *σ*_*j*_^′2^)^2^ = *n*/(*n* + *σ*_*j*_^′2^).

**Remark 8**

By $\sigma_{j}^{\prime 2}\leq {||G_{0}||}_{F}^{2}=n{||\hat{x}||}_{2}^{2}$, we get $1+\left(\sigma_{j}^{\prime 2}/n\right)\leq 1+{||\hat{x}||}_{2}^{2}$, and hence $1/\left(1+{||\hat{x}||}_{2}^{2}\right)\leq n/\left(n+\sigma_{j}^{\prime 2}\right)$.

**Algorithm 9**

 
* *

*Step  1*. Form the block style spectral decomposition of the matrix *C*

(55) $$\begin{array}{c} C=Q\left(\begin{array}{ccc} C_{1} & & \\ & \ddots & \\ & & C_{t} \end{array}\right)Q^{T}. \end{array}$$

*Step  2*. Compute $r=b-A\hat{x}$.

*Step  3*. Compute *r*_0_ = *Q* ^*T*^ *r*.

*Step  4*. Compute $Q^{T}\hat{x}=\left(\begin{array}{c} x_{1}^{\left(0\right)}\\ \vdots \\ x_{1}^{\left(0\right)} \end{array}\right)$.

*Step  5*. Form *G*_0_.

*Step  6*. Compute $\eta^{2}(\hat{x})$.

## 4. Conclusion

 The related problems of skew-circulant matrix are considered in this paper. We not only present style spectral decomposition and singular value but also study backward perturbation analysis for the linear system with skew-circulant coefficient matrix. The reason why we focus our attentions on skew-circulant is to explore the application of skew circulant in the related field in medicine. Wittsack et al. in [16] validated a deconvolution method originating from magnetic resonance techniques and apply it to the calculation of dynamic contrast enhanced computed tomography perfusion imaging, and the application of a block circulant matrix approach for singular value decomposition renders the analysis independent of tracer arrival time to improve the results. On the basis of existing application situation, we conjecture that SVD decomposition of skew circulant matrix will play an important role in CT-perfusion imaging of human brain.
